# Mechanistic Insights into the Combined Anti-inflammatory Effects of Bevacizumab and Triamcinolone in Hyperglycemic Retinal Cells: Modulation of MCP-1, CXCL10, and Their Receptors

**DOI:** 10.18502/jovr.v21.19302

**Published:** 2026-09-03

**Authors:** Zahra Souri, Mohammad Malekahmadi, Farzad Pakdel

**Affiliations:** ^1^Department of Cell and Molecular Biology & Microbiology, Faculty of Biological Science and Technology, University of Isfahan, Isfahan, Iran; ^2^Isfahan Eye Research Center, Department of Ophthalmology, Isfahan University of Medical Sciences, Isfahan, Iran; ^3^Department of Oculo-Facial Plastic Surgery, Farabi Eye Hospital, Faculty of Medicine, Tehran University of Medical Sciences, Tehran, Iran; ^4^Craniomaxillofacial Research Center, Shariati Hospital, Tehran University of Medical Sciences, Tehran, Iran

**Keywords:** Bevacizumab, Diabetic Retinopathy, Inflammation, Retinal Pigment Epithelium, Triamcinolone

## Abstract

**Purpose:**

Diabetic retinopathy is marked by hyperglycemia-induced inflammation and chemokine signaling that drive immune cell infiltration and retinal damage. As bevacizumab offers limited benefit in some cases, targeting broader inflammatory pathways with agents like triamcinolone may enhance therapeutic outcomes.

**Methods:**

ARPE-19 cells exposed to hyperglycemic conditions (30 mM glucose) for 72 hours were treated with bevacizumab (0.3 mg/ml), triamcinolone (1mg/ml), or their combination. Cell proliferation was assessed using the MTT assay, while ELISA measured VEGF, MCP-1, and CXCL10 secretion, and qRT-PCR analyzed *CCR2* and *CXCR3* gene expression. Three independent biological replicates were used in each experiment.

**Results:**

Hyperglycemia markedly increased cell proliferation after 24 and 48 hours (*P* < 0.001). Bevacizumab or triamcinolone alone did not alter cell proliferation under hyperglycemic stress; however, their combined treatment significantly reduced proliferation (*P* < 0.001). Hyperglycemia markedly elevated VEGF secretion (*P* < 0.001), whereas bevacizumab, triamcinolone, and especially their combination significantly suppressed it (all *P* < 0.05). All three treatment conditions significantly reduced MCP-1 levels (*P* < 0.05), while only the combined therapy lowered CXCL10 secretion (*P* < 0.01). Regarding chemokine receptors, both drugs individually downregulated *CCR2* and *CXCR3* expression, with the combination producing an additive inhibitory effect (*CCR2*: *P* < 0.001; *CXCR3*: *P* < 0.05).

**Conclusion:**

These results reveal that triamcinolone may complement bevacizumab by targeting VEGF-independent inflammatory pathways, providing mechanistic insight into combination therapy for diabetic macular edema (DME), particularly in patients with persistent DME despite anti-VEGF monotherapy.

##  INTRODUCTION

Diabetic retinopathy (DR) is the most common microvascular complication of diabetes mellitus (DM) and remains a leading cause of vision loss worldwide, affecting nearly 33% of individuals with DM.^[1]^ Approximately 537 million adults aged 20 to 79 are living with DM, representing 10.5% of the global population, and this prevalence is projected to rise beyond 12% by 2045.^[2]^ DR arises from chronic hyperglycemia, often leading to irreversible damage to the retinal microvasculature. This pathological cascade leads to retinal ischemia, inflammation, vascular leakage, and ultimately vision-threatening complications such as diabetic macular edema (DME).^[3,4]^ At present, inhibition of vascular endothelial growth factor (VEGF) is the first-line treatment for DME. While VEGF is a well-established driver of vascular permeability in DME, accumulating evidence indicates that DR is not solely a vascular disease but also a chronic inflammatory disorder.^[5,6,7]^

Monocyte chemoattractant protein-1 (MCP-1) is a cytokine that plays a pivotal role in the progression of DR by recruiting monocytes and macrophages into the retina, thereby promoting sustained inflammation and vascular damage. MCP-1 acts primarily through its receptor CCR2, which contributes to leukocyte infiltration, neovascularization, and blood retinal barrier (BRB) disruption.^[8,9]^. Similarly, C-X-C motif chemokine ligand 10 (CXCL10) interacts with its receptor CXCR3 to orchestrate recruitment and activation of T cells and natural killer cells, further amplifying retinal inflammation.^[10,11]^ Together, the MCP-1/CCR2 and CXCL10/CXCR3 axes are increasingly recognized as central mediators of chronic inflammation in DR and may contribute to therapeutic resistance observed in some patients. Intravitreal pharmacological agents remain the cornerstone of DME management. Bevacizumab, a monoclonal antibody targeting VEGF, reduces vascular permeability, retinal edema, and neovascularization, with its clinical efficacy in DME and proliferative DR supported by multiple trials.^[12,13,14]^

Despite the proven efficacy of anti-VEGF bevacizumab for DME, a considerable proportion of patients demonstrate suboptimal or incomplete responses, highlighting that VEGF-driven leakage is not the sole mechanism underlying disease progression; moreover, some side effect profiles such as delayed inflammation or gliosis may occur.^[15,16]^ Triamcinolone, a corticosteroid, offers broader anti-inflammatory activity by suppressing multiple cytokines, stabilizing the BRB, and reducing vascular leakage. It is particularly effective in chronic or treatment refractory DME cases.^[17,18]^

Most studies have investigated anti-VEGF or corticosteroid therapy separately and have not examined how dual targeting of angiogenesis and inflammation influences key chemokine pathways. Therefore, their regulation by combined bevacizumab/triamcinolone therapy warrants further research. Using ARPE-19 cells as a relevant *in vitro* model of diabetic retinal inflammation, this study evaluates whether dual therapy more effectively modulates MCP-1/CCR2 and CXCL10/CXCR3 axes under hyperglycemic stress, providing mechanistic insight that may help explain treatment resistance in DME.

##  METHODS 

### Cell Culture and Reagents

The human retinal pigment epithelial cell line (ARPE-19) was purchased from Royan Institute for Biotechnology, Iran. ARPE-19 cells were cultured in Dulbecco's Modified Eagle's Medium (DMEM): F12 medium (Bioidea, Iran), supplemented with 10% fetal bovine serum (FBS) and 1% penicillin/streptomycin (Bioidea, Iran). The cells were maintained at 37ºC in a 5% CO ${}_{2}$ atmosphere with 90% relative humidity.

### Glucose Treatment

Upon reaching 80% confluence, cells were subcultured and incubated in DMEM media (Bioidea, Iran) supplemented with either 5.5 mM normal glucose (NG) or 30 mM high glucose (HG; D-Glucose; Sigma-Aldrich, St. Louis, Missouri, USA) for 72 hours. To exclude any potential bias due to osmotic effect, cells were treated with 24.5 mmol/L mannitol (Sigma-Aldrich). The plates were maintained in a humidified CO ${}_{2}$ incubator at 37ºC with 5% CO ${}_{2}$ .

### Bevacizumab and Triamcinolone Treatment 

After 72 hours of glucose exposure, ARPE-19 cells were seeded at 2 × 10⁵ cells/well in 6-well tissue culture plates. Treatments were applied as follows.

• Control groups: Cells cultured in DMEM containing either NG or HG.
• Bevacizumab group (B): Cells treated with bevacizumab 0.3 mg/mL (Stivant, Aryogen Pharmed, Iran) for 48 hours, which is compatible with the clinically effective concentration inside the eye, assuming a vitreous volume of approximately 4 mL in adults.
• Triamcinolone group (T): Cells treated with triamcinolone 1 mg/mL (Exir Pharmaceutical Co., Iran) for 48 hours, which reflects the clinically effective concentration inside the eye, assuming a vitreous volume of approximately 4 mL in adults.
• Combination group (BT): Cells treated concurrently with bevacizumab (0.3 mg/mL) and triamcinolone (1 mg/mL) for 48 hours.

### MTT Assay

Cells were seeded in triplicate at 1 × 10^4^ cells/well in 96-well plates. After 48 hours of either treatment with bevacizumab or triamcinolone or their combination, or no treatment, the metabolic activity of the cultured ARPE-19 cells was assessed using the MTT assay. A medium containing 0.5 mg/mL MTT (Sigma-Aldrich) was added to each well and incubated for 4 hours at 37ºC. Following incubation, the medium was removed, and 100 μ L of DMSO (Sinaclon, Iran) was added to dissolve the formazan crystals formed by MTT cleavage. Optical absorbance at 570 nm was then measured using a Stat Fax-2100 microplate reader (Awareness Technology, Inc, Palm City, Florida, USA). The experiment was performed in triplicate, and the results were expressed as a percentage of cell viability.

### Quantitative Real-time (qRT) PCR

Total RNA was extracted from the cells of each group using TRIzol reagent (MaxZol, Iran). For each group, three independent biological replicates were processed. Equal amounts of RNA were then converted to cDNA using the cDNA synthesis Kit (Yekta Tajhiz Azma, Iran) following the manufacturer's protocol, and the cDNA was stored at - 20ºC. Quantitative RT-PCR was performed on the Rotor-Gene Q (QIAGEN, Hilden, Germany) using SYBR Green PCR Master Mix (Amplicon, Odense, Denmark). Relative gene expression was calculated using the 2 ${}^{-\Delta \Delta Ct}$ method and normalized to *HPRT1 *expression.

Data analysis was conducted according to the manufacturer's guidelines. The specific gene primers used are listed in Table 1.

**Table 1:** Primer sequences used for gene expression analysis

**Primer**	**Sequence (5' to 3')**	**Tm**	**Amplicon size**	**NCBI Ref Seq**
CCR2 (Forward) CCR2 (Reverse)	GGAGAAGTTCAGAAGCCTTTTTC GGACCTCCACACACTGGTTTT	58 60	78	NM_001123041.3
CXCR3 (Forward) CXCR3 (Reverse)	TGCCAATACAACTTCCCACA CGGAACTTGACCCCTACAAA	57 57	371	NM_001142797.2
HPRT1 (Forward) HPRT1 (Reverse)	GACCAGTCAACAGGGGACAT CCTGACCAAGGAAAGCAAAG	59 57	132	NM_000194.3

### ELISA Assay

Equal numbers of RPE cells (2 × 10^5^) were seeded in each well of six-well plates and cultured in identical medium volumes (2 mL per well). Cell culture medium supernatants were collected after 48 hours of drug treatment, centrifuged at 1000 × g for 10 minutes to remove debris, and stored at - 80ºC until analysis. Secretion levels of VEGF, MCP-1, and CXCL10 (pg/mL) were measured using enzyme-linked immunosorbent assay kits (Karmania Pars Gene, Iran) according to the manufacturer's instructions. The detection sensitivity for all three cytokines was 2 pg/mL. For each cytokine, 100 µL of supernatant was loaded per well. Absorbance was measured at 450 nm using a microplate reader, and concentrations were determined from standard curves. As all wells contained equal cell numbers and medium volumes, cytokine concentrations were expressed directly as pg/mL of culture medium.

### Statistical Analysis

Experiments were performed in triplicate and repeated across three independent biological samples to ensure reproducibility. Data were analyzed with SPSS version 22.0 (IBM Corp., Armonk, New York, USA), and graphs were generated using GraphPad Prism version 5.0 (GraphPad Software, La Jolla, California, USA). Differences among multiple groups were assessed using one-way ANOVA, followed by Tukey's post-hoc test for pairwise comparisons, while comparisons between two independent groups were analyzed using an independent *t*-test. Statistical significance was indicated as *P* ≤ 0.05 (*)*, P ≤ 0.01 *(**)*, and P ≤ 0.001 *(***).

**Figure 1:**
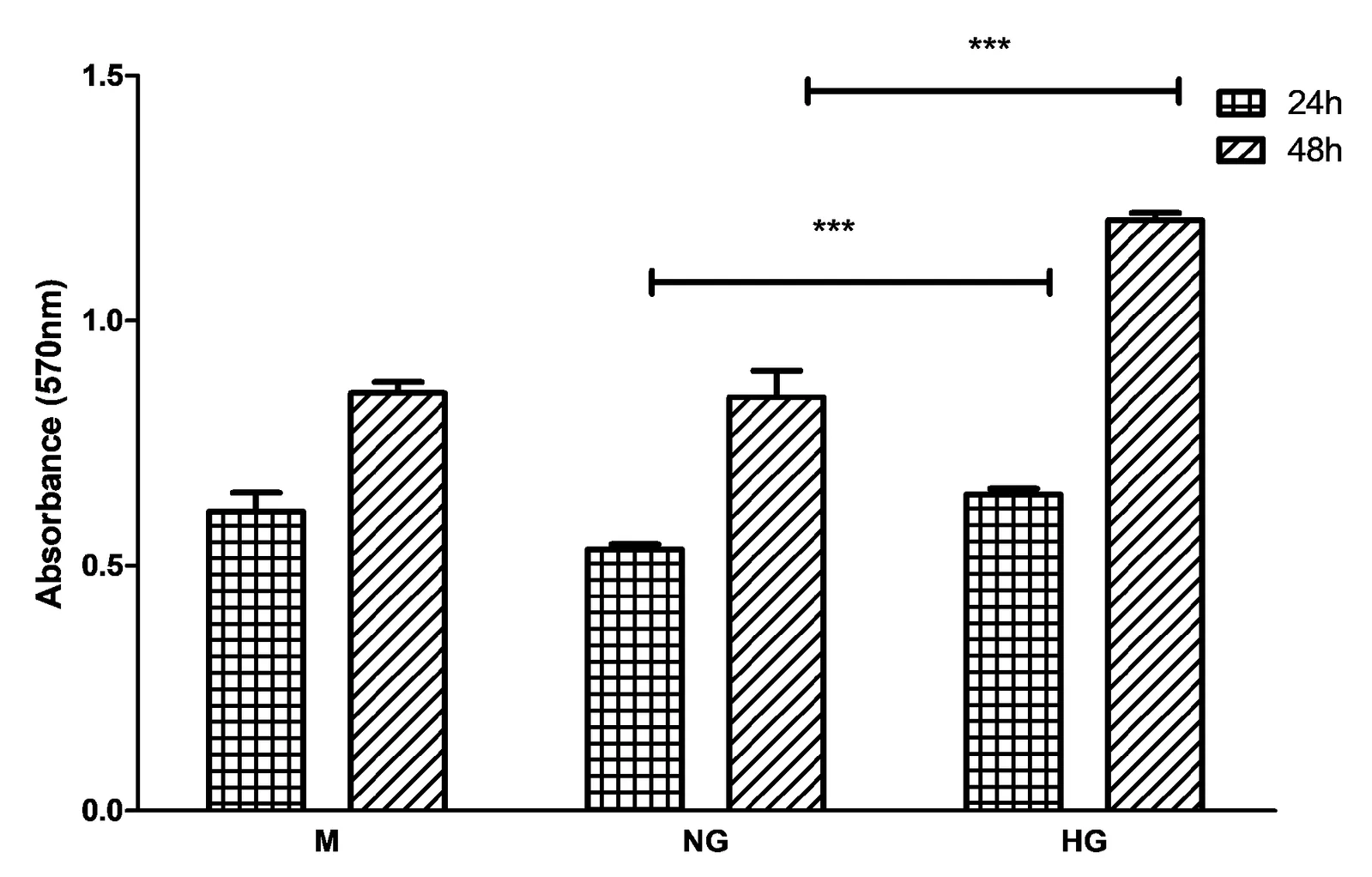
Proliferation of ARPE-19 cells in normal glucose (5.5 mM) versus high glucose (30 mM) conditions after 24 and 48 hours of treatment. Cell density was measured using an MTT assay. Data are shown as mean ± SEM of three independent biological replicates. Statistical significance was assessed by independent *t*-test: *P* ≤ 0.05 (*), *P* ≤ 0.01 (**), *P* ≤ 0.001 (***). M, mannitol osmotic control; NG, normal glucose; HG, high glucose.

**Figure 2:**
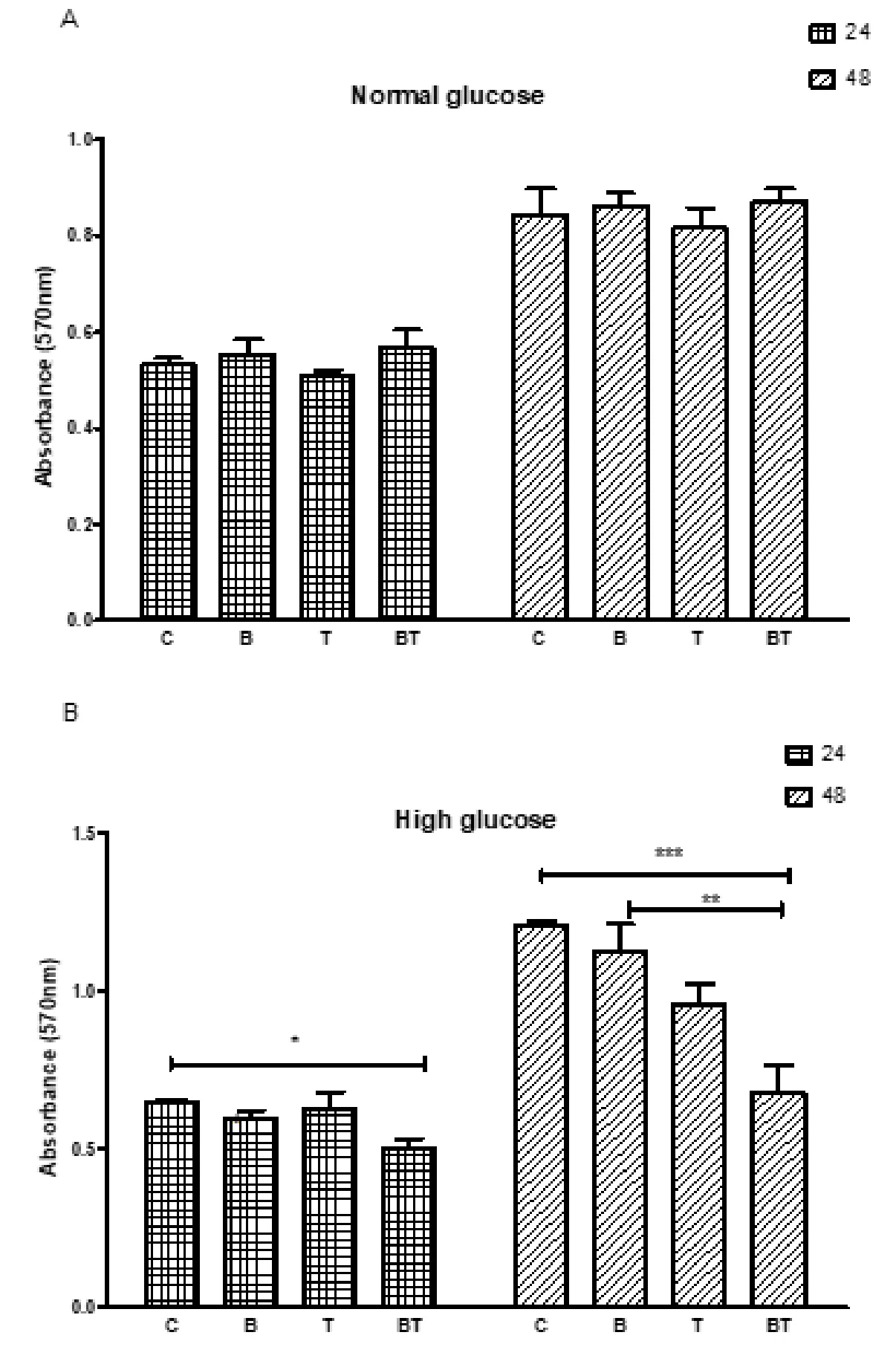
Proliferation of ARPE-19 cells in (A) normal glucose (5.5 mM) and (B) high glucose (30 mM) conditions, following treatment with bevacizumab (0.3 mg/mL), triamcinolone (1 mg/mL), and their combination (0.3 mg/mL B + 1 mg/mL T) at 24 and 48 hours. Cell density was measured using an MTT assay. Data are shown as mean ± SEM of three independent biological replicates. Statistical significance was assessed using one-way ANOVA with Tukey's post-hoc test: *P* ≤ 0.05 (*), *P* ≤ 0.01 (**), *P* ≤ 0.001 (***). C, control; B, bevacizumab; T, triamcinolone; BT, combination.

##  RESULTS

### Effect of Hyperglycemia on Cell Proliferation 

To investigate the effect of hyperglycemia on RPE cell behavior, we cultured ARPE-19 cells under NG and HG conditions and assessed their proliferation over time [Figure 1]. At 24 hours, cells exposed to HG exhibited significantly increased proliferation compared to the NG group (0.53 ± 0.01 vs 0.64 ± 0.01, *P* = 0.005).

A similar pattern was observed after 48 hours (0.84 ± 0.05 vs 1.20 ± 0.01, *P *= 0.007). Importantly, no significant difference in proliferation was noted between the mannitol and control groups (*P* > 0.05), confirming that the effect was glucose-specific rather than due to osmotic stress.

### Effect of Bevacizumab and Triamcinolone on Cell Proliferation in Normal and High Glucose Environments

To evaluate the effect of pharmacologic interventions on ARPE-19 proliferation under varying metabolic conditions, we treated cells cultured in NG and HG media with bevacizumab, triamcinolone, or their combination. In the NG condition (5.5 mM glucose), no significant changes in proliferation were observed across any of the treatment groups (B, T, BT) compared to the untreated NG control at either 24 or 48 hours (*P* < 0.05) [Figure 2A].

In contrast, a divergent response was observed under hyperglycemic conditions (30 mM glucose). At 24 hours, combination therapy (BT) significantly reduced cell proliferation compared to the HG control group (0.64 ± 0.02 vs 0.50 ± 0.06, *P* = 0.04). This effect became more prominent at 48 hours, with BT leading to significant reductions in proliferation compared to both untreated cells and bevacizumab alone (1.2 ± 0.02 vs 0.67 ± 0.18, *P* < 0.001 and 1.12 ± 0.16 vs 0.67 ± 0.18, *P* < 0.01, respectively) [Figure 2B]. Notably, bevacizumab monotherapy (B) did not significantly alter proliferation compared to untreated cells at either time point.

### Effect of Bevacizumab and Triamcinolone on VEGF, MCP-1, and CXCL10 Secretion in Normal and High Glucose Environments

To assess inflammatory responses under different glucose and treatment conditions, the levels of VEGF, MCP-1, and CXCL10 were quantified in the supernatants of ARPE-19 cells using ELISA after 48 hours of exposure. Cytokine profiles varied distinctly based on glucose concentration and treatment modality. The HG group exhibited significantly elevated levels of VEGF compared to the NG group (10.96 ± 0.93 vs 19.28 ± 1.52, *P* < 0.001). No significant differences were observed between the mannitol (M) and control groups, indicating that the cytokine upregulation was driven by metabolic rather than osmotic stress.

Treatment with bevacizumab, triamcinolone, or their combination effectively reduced VEGF secretion in NG cells (3.55 ± 0.2, *P* < 0.01; 6.93 ± 0.29, *P* < 0.05; 7.82 ± 0.96, *P* < 0.01, respectively). A similar pattern was observed in the HG group (13.29 ± 1.24, *P* < 0.05; 8.85 ± 0.78, *P* < 0.01; 14.52 ± 0.49, *P* < 0.05, respectively) [Figure 3A].

However, MCP-1 levels displayed treatment-specific modulation. Compared to untreated cells, bevacizumab alone did not significantly affect MCP-1 secretion under NG (71.35 ± 3.74 vs 91.84 ± 12.25, *P* > 0.05) and HG (93.25 ± 6 vs 95.72 ± 11.99, *P* > 0.05) conditions. In contrast, triamcinolone significantly suppressed MCP-1 levels in HG environments after 48 hours (51.42 ± 6.61, *P* < 0.05), while reduced MCP-1 compared to bevacizumab alone in NG (91.84 ± 12.25 vs 40.20 ± 11.27, *P* < 0.05) and HG (95.72 ± 11.99 vs 51.42 ± 6.66, *P* < 0.05) conditions. Combination therapy suppressed MCP-1 in both NG (33.91 ± 3.4, *P* < 0.05) and HG (39.49 ± 4.76, *P* < 0.01) conditions and further reduced MCP-1 secretion compared to bevacizumab monotherapy in those conditions (*P* < 0.05, *P* < 0.05) [Figure 3B].

Combination therapy significantly reduced CXCL10 secretion compared to untreated cells under both NG (19.76 ± 2 vs 3 ± 1.4, *P* < 0.01) and HG (27.36 ± 1.26 vs 3.48 ± 2, *P* < 0.01) conditions, as well as compared to bevacizumab and triamcinolone monotherapies (*P* < 0.01 for both) [Figure 3C].

### Effect of Bevacizumab and Triamcinolone on CCR2 and CXCR3 mRNA Expression in Normal and High Glucose Environments

To investigate how hyperglycemia and drug treatments affect chemokine receptor signaling pathways, we measured the mRNA expression levels of CCR2 and CXCR3 in ARPE-19 cells cultured under NG and HG conditions using qPCR [Figures 4A–4D]. Gene expression was normalized to the untreated NG control, which was set to 1. Expression levels in the mannitol (M) group did not significantly differ from those in the NG control, confirming that changes in CCR2 and CXCR3 expression were attributable to glucose-induced metabolic stress rather than osmotic effects. Under NG conditions, bevacizumab selectively reduced CXCR3 expression (Fold change, *P* < 0.05) but did not significantly affect CCR2. In contrast, both triamcinolone and combination therapy significantly downregulated CCR2 (Fold change 0.002, *P* < 0.01; Fold change 0.005, *P* < 0.01) and CXCR3 (Fold change 0.39, *P* < 0.01; Fold change 0.27, *P* < 0.01) expression [Figures 4A & 4B].

In the HG group, all three treatment modalities, including bevacizumab (Fold change 0.0025), triamcinolone (Fold change 0.00033), and their combination (Fold change 0.00075), significantly suppressed CCR2 expression (all *P* < 0.001). In contrast, CXCR3 expression was only significantly reduced by bevacizumab (Fold change 0.44) and the combination therapy (Fold change 0.36, both *P* < 0.05) [Figures 4C & 4D].

**Figure 3:**
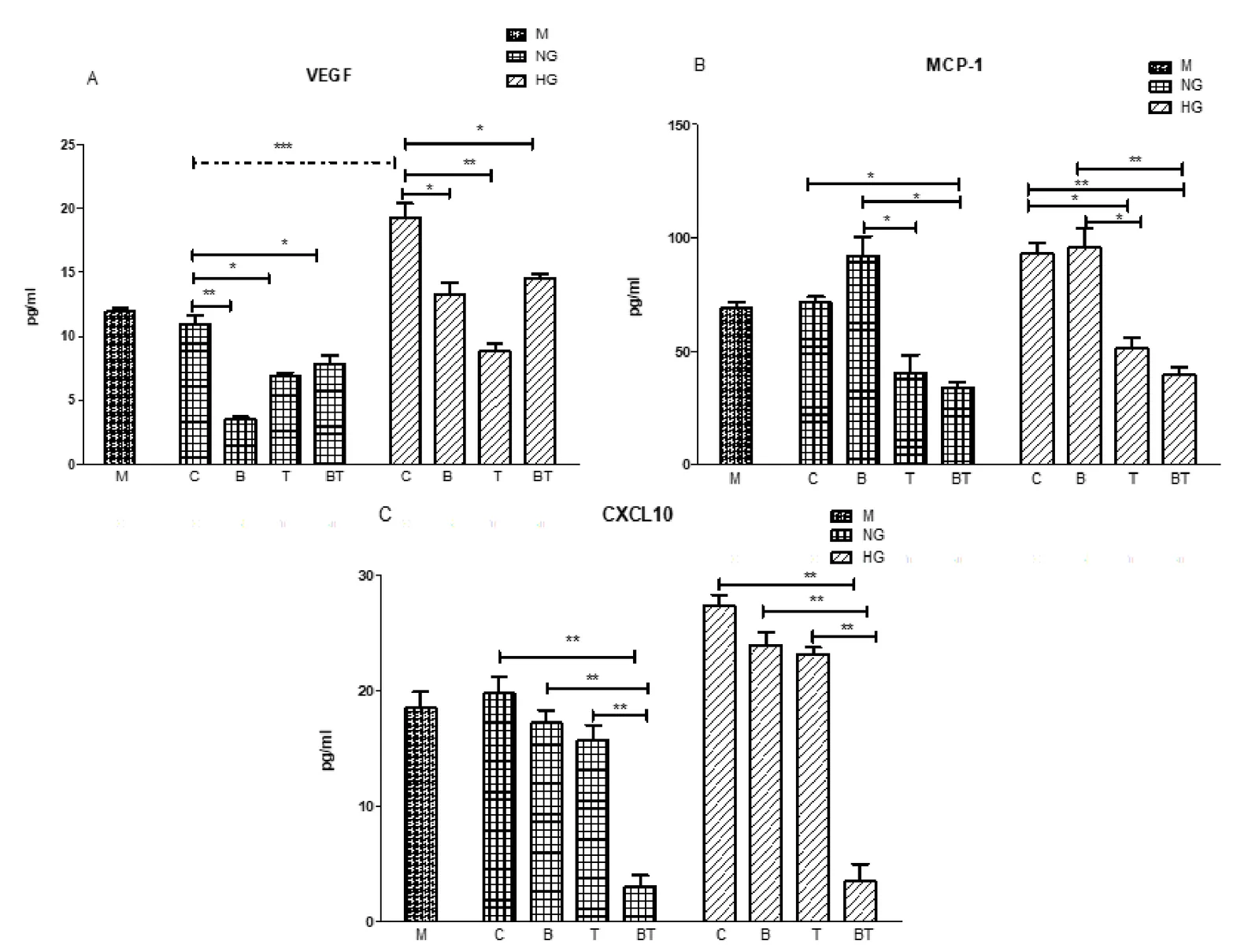
(A) VEGF, (B) MCP-1, and (C) CXCL10 secreted protein levels in ARPE-19 cell culture supernatants in normal glucose (5.5 mM) and high glucose (30 mM) conditions, following treatment with bevacizumab (0.3 mg/mL), triamcinolone (1 mg/mL), and their combination (0.3 mg/mL + 1 mg/mL) for 48 hours. Data are shown as mean ± SEM of three independent biological replicates. Statistical significance was assessed using one-way ANOVA with Tukey's post-hoc test: *P* ≤ 0.05 (*), *P* ≤ 0.01 (**), *P* ≤ 0.001 (***). M, mannitol osmotic control; C, control; B, bevacizumab; T, triamcinolone; BT, combination.

**Figure 4:**
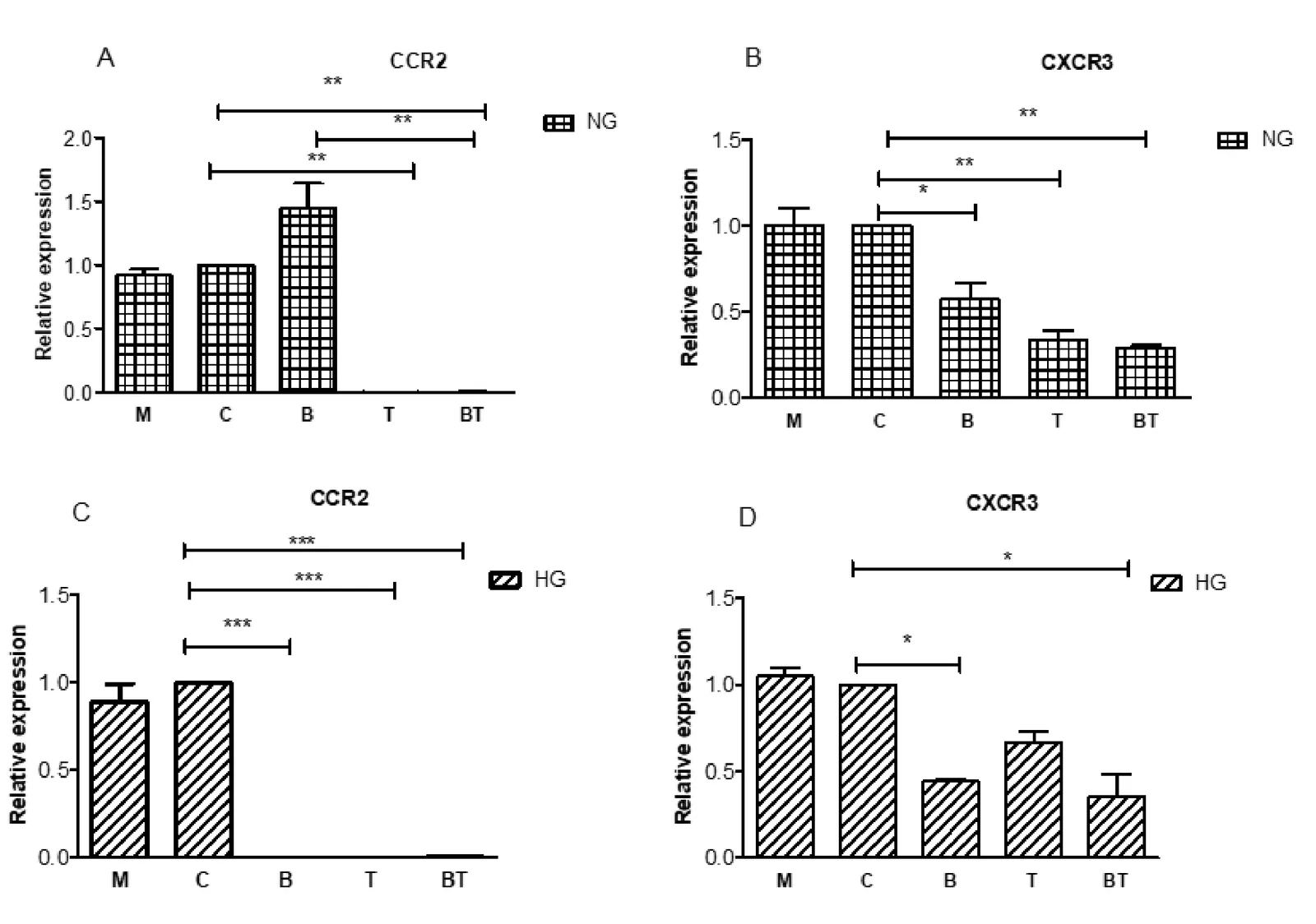
Relative CCR2 and CXCR3 mRNA expression in ARPE-19 cells in (A & B) normal glucose (5.5 mM) and (C & D) high glucose (30 mM) conditions, following treatment with bevacizumab (0.3 mg/mL), triamcinolone (1 mg/mL), and their combination (0.3 mg/mL + 1 mg/mL) for 48 hours. Data are shown as mean ± SEM of three independent biological replicates. Statistical significance was assessed using one-way ANOVA with Tukey's post-hoc test: *P* ≤ 0.05 (*), *P* ≤ 0.01 (**), *P* ≤ 0.001 (***). M, mannitol osmotic control; C, control; B, bevacizumab; T, triamcinolone; BT, combination.

##  DISCUSSION

This work gives preliminary insights into the interaction between hyperglycemia-induced inflammation and bevacizumab or triamcinolone therapy in ARPE-19 cells, indicating that the biological imbalance in the immune system under stress might play a role in different or persistent responses to therapy in patients with DR.

Hyperglycemia has been known to cause abnormal cellular functions in the retina. In addition, it has been indicated that if the retina is exposed to a high glucose concentration, it leads to increased expression of iNOS, COX-2, and leukotrienes.^[19,20]^ Our results align with the fact that high glucose concentration stimulates the proliferation of RPE cells. Hyperproliferation has been implicated in the progression of DR through BRB disruption.^[21,22]^

The prominent factor known to mediate neo-angiogenesis and enhance vascular permeability under hypoxia in DR is VEGF.^[23,24]^ VEGF is recognized as a cause of inflammation in the disease, resulting in the activation of leukocytes and further contributing to the elevation of pro-inflammatory cytokines.^[25,26]^

Anti-VEGF bevacizumab has been used extensively as the primary option to treat macular edema however;^[27]^ it has been observed that up to two-thirds of patients require conversion to other forms of therapy because of resistance to bevacizumab.^[28,29,30]^ Triamcinolone was among the primary corticosteroids that were injected intravitreally for persistent DME and resulted in visual recovery.^[31]^

**Figure 5:**
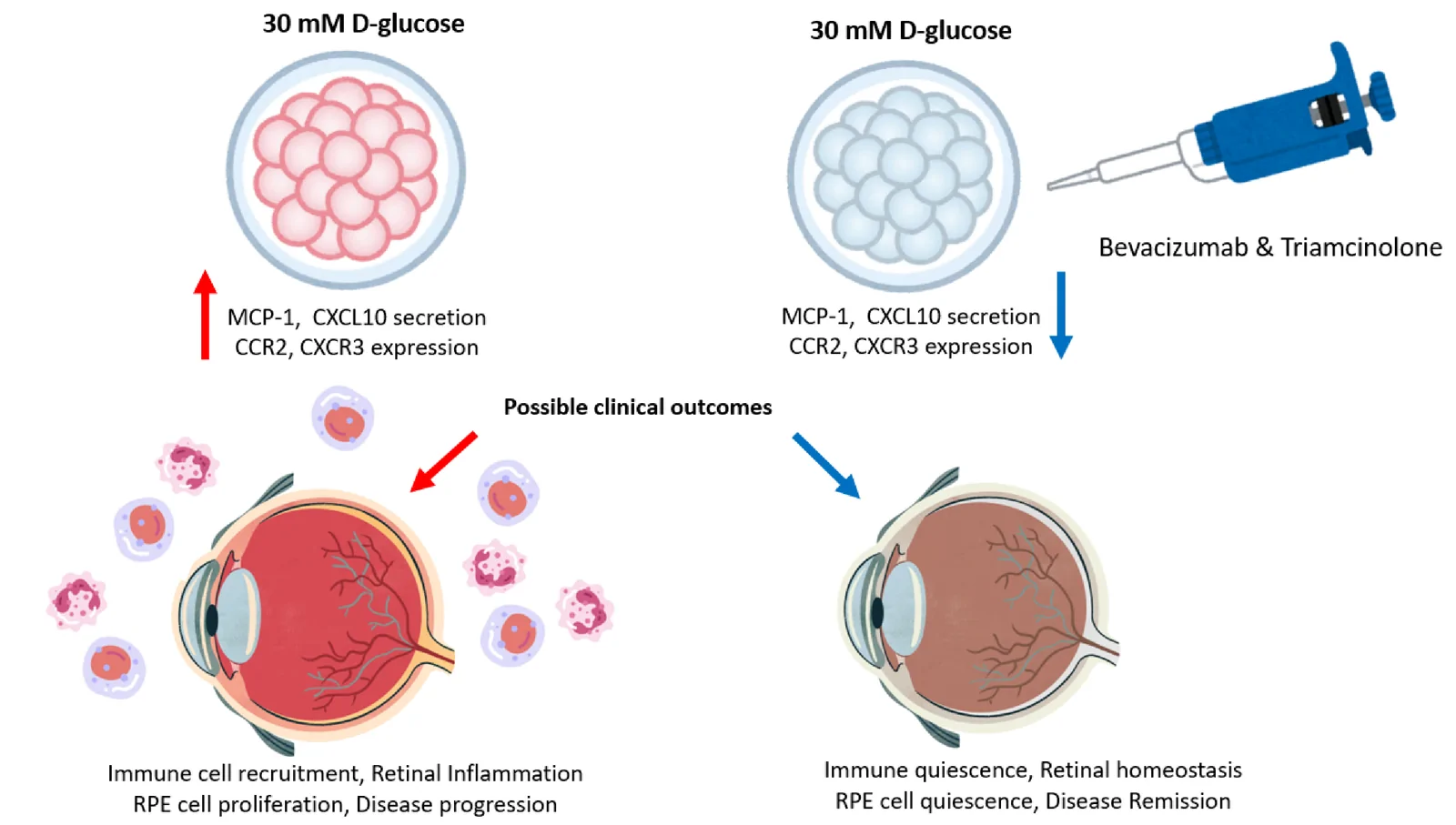
Conceptual model summarizing the *in vitro* immunomodulatory effects of bevacizumab and triamcinolone in hyperglycemic RPE cells. High glucose (30 mM) increased MCP-1, CXCL10, CCR2, and CXCR3 expression, indicating inflammatory activation. Treatment with bevacizumab and triamcinolone reduced these markers, suggesting a shift toward immune suppression. This schematic represents a conceptual summary derived from *in vitro* findings, not direct mechanistic evidence, and illustrates potential pathways through which these agents may reduce retinal inflammation in diabetic eye disease.

In our study, we applied a combination therapy of bevacizumab and triamcinolone and found that it resulted in significantly lower proliferation in ARPE-19 cells under hyperglycemic conditions, but no significant effect under normal conditions. The finding is consistent with earlier works that suggest metabolic stress increases the susceptibility of retinal cells to a pharmacological agent, implying that a hyperglycemic microenvironment is pivotal in mediating drug response.^[32]^

In accordance with the current literature, VEGF showed an elevated secretion in hyperglycemic RPE cells, suggesting an angiogenic milieu in DR pathology.^[33]^ Bevacizumab significantly lowered the secretion of VEGF in NG and HG cells. The efficacy of bevacizumab in preventing VEGF-mediated vascular permeability is recognized and well-supported in the literature.^[34]^ Bevacizumab did not show any significant decrease in the secretion of MCP-1 and CXCL-10 in single agent analysis. However, triamcinolone significantly lowered the secretion of VEGF in NG and HG cells and MCP-1 in hyperglycemic cells. The combination of bevacizumab and triamcinolone significantly lowered the secretion of VEGF, MCP-1, and CXCL-10 in NG and HG cells.

The implications of the aforementioned findings are that although bevacizumab efficiently inhibits the VEGF-mediated pro-angiogenic signaling pathway, it cannot adequately address the inflammatory response mediated by hyperglycemia. In this dual therapy, triamcinolone appears to be the key drug by virtue of its anti-inflammatory properties. The complementary inhibition of inflammatory and pro-angiogenic signaling pathways suggests that combination therapy may help re-establish cellular homeostasis under hyperglycemic stress in the context of diabetes complications.^[35,36]^ Although this study investigated only the effect of triamcinolone, other corticosteroids, such as dexamethasone, are employed in DME.^[37]^ These agents share anti-inflammatory properties but differ in drug delivery and pharmacokinetics.

MCP-1 and CXCL10 have more recently been recognized to play prominent roles in BRB dysfunction, inflammation, and immunological abnormalities in the diabetic retina.^[38,39]^ The importance of MCP-1 is chiefly in macrophage infiltration of the retina.^[40]^ The concentration in the vitreous has been postulated to be positively correlated with the severity of DR, possibly due to the induction of VEGF.^[41]^ The level of CXCL10, which is an inflammation mediator, is elevated in the aqueous humor of patients with DR and promotes leukocyte adhesion to the retina.^[42]^

The greater chemokine inhibition with the combination therapy suggests a benefit in modulating inflammation independently of VEGF signaling. The increased inhibition of these two chemokines can be related to the reduced expression of CCR2 and CXCR3, which play crucial roles in mediating chemotaxis in MCP-1 and CXCL10, respectively. These are well recognized because of their involvement in monocyte and T-cell trafficking across the BRB, leukostasis, and neovascularization in patients with DR.^[43,44]^

As indicated in Figure 5, our data demonstrate that hyperglycemia primes ARPE-19 cells toward a pro-inflammatory state characterized by increased expression of MCP-1/CXCL10 and their corresponding receptors. Bevacizumab primarily blocks the VEGF-mediated signaling of angiogenesis, while the inhibitory effect of triamcinolone is stronger on inflammation-related chemokinetic signaling. The combination blocks the expression of the receptors and ligands. Therefore, there is a connection between the signaling mechanisms of inflammation and angiogenesis in ARPE-19 cells under hyperglycemia conditions. While targeting VEGF in inflammation offers insights into dual-target effects in a cellular model, one should note that ARPE-19 cells do not perfectly recapitulate human physiology, and pharmacodynamic discrepancies across models may limit direct application of the results to *in vivo* conditions. Indeed, our study provides insights into cellular mechanisms rather than direct evidence of clinical efficacy. Clinical randomized controlled trials evaluating the adjunctive use of intravitreal triamcinolone in combination with anti-VEGF therapy in DME have reported variable outcomes, and in several studies, no clear sustained additional benefit over anti-VEGF monotherapy has been demonstrated. Some reports suggest early functional improvement, whereas longer-term follow-up generally fails to show significant additive anatomical or visual advantage.^[45,46,47,48]^ These findings suggest that variability in clinical response may be linked to underlying inflammatory heterogeneity in DME, emphasizing the need for biomarker-driven approaches to identify patients who may benefit from adjunctive corticosteroid therapy.

In summary, our study provides mechanistic insights into the relationship between hyperglycemia-associated inflammation and subsequent pharmacotherapy with RPE cells. Bevacizumab effectively reduces the amount of secreted VEGF, but has little effect on the levels of pro-inflammatory chemokines MCP-1 and CXCL10. Triamcinolone, in a manner complementary to the inhibition of VEGF, downregulates chemokine levels and their respective receptors, CCR2 and CXCR3. This highlights a dual process that links angiogenic and inflammatory pathways. The relationship we described under hyperglycemic conditions may reflect a biological variability in responses to anti-VEGF therapy in diabetic patients, while supporting MCP-1, CXCL10, and their host receptors as potential mechanistic biomarkers for phenotyping DME. To substantiate these observations further, in vivo and clinical studies are warranted to validate MCP-1 and CXCL10 as predictive biomarkers of response to combined anti-VEGF and corticosteroid therapy.

##  Financial Support and Sponsorship

This study was funded by the University of Isfahan.

##  Conflicts of Interest

None.
